# Integrating anisotropic heat flow and transformer encoders in convolutional neural network for skin cancer classification

**DOI:** 10.3389/fmed.2026.1834696

**Published:** 2026-06-09

**Authors:** Sanad Aburass, Osama Dorgham, Ibrahim Aljarah

**Affiliations:** 1 Department of Computer Science, Luther College, Decorah, IA, United States; 2 Prince Abdullah bin Ghazi Faculty of Information and Communication Technology, Al-Balqa Applied University, Al-Salt, Jordan; 3 Department of Artificial Intelligence, The University of Jordan, Amman, Jordan

**Keywords:** Advanced Heat Flow, anisotropic diffusion, deep learning, dermatological diagnostics, medical imaging, skin cancer classification

## Abstract

The early detection and classification of skin cancer are pivotal in improving patient outcomes and reducing healthcare burdens. However, traditional deep learning models in dermatological diagnostics often struggle with the nuanced differentiation of skin lesions. This paper introduces an approach to integrate an Advanced Heat Flow Layer into deep learning architectures for skin cancer classification, this method is centered on the principles of anisotropic diffusion, distinguishing itself from conventional image processing techniques by selectively smoothing image areas while preserving critical edge details, essential for accurate lesion identification. In our research, we utilized the Ham10000 dataset, enriched with data augmentation to simulate real-world variability, we conducted a comprehensive comparison of our model, featuring the Advanced Heat Flow Layer, against several benchmark deep learning models, including Sobel Edge Detection Layer. Our model, integrated with various layers of DenseNet121, consistently outperformed these benchmarks across key metrics such as accuracy, precision, recall, F1 score, and AUC, particularly with augmented data, this indicates a significant enhancement in the model’s ability to generalize and maintain critical diagnostic features under diverse conditions. Our code is available at, https://github.com/sanadv/SkinCancerClassificationModels/blob/main/Models.ipynb

## 1 Introduction

The field of medical image processing has undergone a paradigm shift with the advent of machine learning (ML) and deep learning (DL) techniques (1–3). Skin cancer, notably melanoma, basal cell carcinoma, and squamous cell carcinoma, poses diagnostic challenges due to its diverse morphological characteristics, this necessitates advanced computational approaches for accurate classification and early detection, directly impacting patient prognosis and treatment efficacy (4–7). The integration of ML in dermatological imaging has been pivotal in transcending the limitations of traditional diagnostic modalities, offering enhanced specificity and sensitivity in lesion analysis, deep learning, especially through the utilization of Convolutional Neural Networks (CNNs), has emerged as a cornerstone in medical imaging, demonstrating proficiency in extracting hierarchical features from complex image datasets (8–11). The inception of architectures like DenseNet121 has furthered this capability, providing an efficient framework for feature extraction and propagation in deep networks, DenseNet’s unique architecture facilitates feature reuse at each layer, enhancing the flow of information and gradients throughout the network, which is particularly advantageous in the context of high-dimensional medical images (12–15).

Our study introduces a new approach, integrating DenseNet121 with a custom-designed Advanced Heat Flow Layer and transformer encoder layers, to address the intricate challenges in skin cancer classification, the proposition of the Advanced Heat Flow Layer is a significant divergence from traditional image preprocessing methods in dermatological imaging, this layer employs anisotropic diffusion, a technique rooted in partial differential equations, to perform edge-preserving image smoothing, Anisotropic diffusion is particularly adept at enhancing image quality by attenuating noise while preserving crucial edge information—a feature paramount in accurately delineating lesion boundaries and characteristics (16). Furthermore, recognizing the spatial complexity of dermatological images, our model integrates transformer encoder layers. Vision Transformers (ViT), renowned for their success in sequence modeling and natural language processing, are adapted in our approach to handle the spatial dimensionality of skin lesion images, the application of self-attention mechanisms within these layers allows the model to weigh and integrate information across the entire image, enabling it to capture long-range dependencies and subtle patterns critical for lesion classification (17–19).

The efficacy of our approach is rigorously evaluated using the HAM10000 dataset, a large-scale, publicly available collection of dermatoscopic images, annotated with a diverse range of skin lesions, this dataset, encompassing over 10,000 images, provides a comprehensive and heterogeneous mix of lesion types and patient demographics, it is a pertinent choice for model validation, presenting a realistic spectrum of clinical scenarios and ensuring the generalizability and robustness of our approach (20).

In addition to the aforementioned methodologies, a critical aspect of our approach is the application of Ensemble Learning, a technique that has demonstrated substantial improvements in predictive performance across various domains, including medical image analysis, Ensemble Learning involves the integration of multiple models or algorithms to achieve better predictive accuracy than could be obtained from any of the individual models alone (21, 22). In the context of our study, we leverage this principle by combining the outputs of multiple distinct models each informed by DenseNet121, the Advanced Heat Flow Layer, and transformer encoder layers, this ensemble approach is designed to exploit the complementary strengths of each model variant, thereby enhancing the robustness and reliability of our skin cancer classification system.

The rationale behind adopting Ensemble Learning is rooted in the notion that different models may capture diverse aspects of the data, leading to a more comprehensive understanding of the complex patterns present in dermatoscopic images, by aggregating the predictions from multiple models, we aim to mitigate the risk of individual model biases or overfitting, common challenges in deep learning-based medical image analysis (23, 24). The ensemble’s collective decision, typically achieved through methods such as voting or averaging, is expected to offer a more balanced and accurate classification outcome, this diversity is a key component of the ensemble’s effectiveness, as it enables the system to cover a broader spectrum of features and patterns within the skin lesion images.

Although anisotropic diffusion and CNN–Transformer architectures are both well established in the literature, the contribution of this work is not to claim either of them as individually new. Rather, this study investigates the integration of a diffusion-inspired Heat Flow Layer within a DenseNet121–Transformer framework for skin cancer classification and evaluates its effect relative to a Sobel-based alternative on the HAM10000 dataset.

In this sense, the proposed contribution is positioned as an application-specific integration of diffusion-inspired processing within a deep skin lesion classification architecture, rather than as a new diffusion theory. While diffusion-based enhancement and PDE-inspired image processing have previously been explored in image analysis, the present study focuses on incorporating such a layer within a DenseNet121–Transformer pipeline and empirically examining its effect on skin cancer classification relative to a Sobel-based alternative.

## 2 Literature review

This literature review explores various approaches, analyzing the strengths and weaknesses of contemporary models in skin lesion identification, it provides a comprehensive overview of the methodologies, datasets, and results from key studies, offering insights into the evolving landscape of AI-driven skin cancer diagnosis.

Zhao examines the performance of CNNs (VGGNet and ResNet) against transformer models (ViT and DeepViT) in classifying skin cancer using the HAM10000 dataset, the research focuses on addressing dataset imbalance through lesion-specific weight adjustments and data augmentation techniques. It concludes that CNNs, specifically VGGNet and ResNet, demonstrate superior accuracy in skin cancer lesion classification compared to the transformer models, highlighting the effectiveness of CNNs in this specific application within medical image analysis (25).

Wu et al., introduce a novel deep neural network, ScATNet, for classifying melanocytic skin lesions in whole slide images (WSIs), the network leverages the concept of scale-awareness, using transformers to handle multi-scale image representations. ScATNet improves performance by focusing on diagnostically relevant information across different scales, the approach includes a soft-labeling method for handling ambiguity in WSIs and demonstrates superior performance compared to several state-of-the-art methods, as well as comparable accuracy to practicing pathologists (26).

Lima and Krohling, focus on evaluating the performance of recent computer vision architectures, particularly CNNs and Transformers, for diagnosing skin lesions, the study emphasizes the use of multimodal feature fusion, integrating image features with clinical data. It conducts extensive experiments on the PAD-UFES-20 dataset, showing that Transformer-based models can achieve competitive performance against CNN-based models in skin lesion diagnosis, the paper also investigates the impact of recent training methods and architectures on the task’s performance (27).

Nie et al., introduce a hybrid model that combines CNNs with ViTs for classifying skin lesions. It employs Focal Loss to address the class imbalance in the ISIC 2018 dataset. The model starts with a ResNet-50 based CNN for feature extraction, followed by a transformer for global feature modeling. This hybrid approach aims to leverage the strengths of both CNNs and transformers in capturing local and global image features for improved classification performance (28).

Xin et al., present a novel ViT based network, SkinTrans, for classifying dermoscopic skin cancer images, it employs multi-scale image serialization and patch embedding to focus on multi-scale features, and incorporates contrastive learning to enhance feature differentiation. The model’s performance is evaluated on two datasets, HAM10000 and a clinical dataset, achieving high accuracy, the study emphasizes the efficiency of ViT in skin cancer classification and its potential for multimodal data application (29).

Bassel et al., propose a stacked classifier model for melanoma and benign skin cancer identification, it employs feature extraction methods like ResNet50, Xception, and VGG16, and uses metrics such as accuracy, F1 scores, AUC, and sensitivity for performance evaluation. The system undergoes training with a dataset of 1,000 skin images, using deep learning, SVM, RF, NN, KNN, and logistic regression methods, the paper emphasizes the improvement of skin cancer classification through a comprehensive, hybrid deep learning approach (30).

Aladhadh et al., present a novel framework for skin cancer classification using the Medical Vision Transformer (MVT), the approach involves extensive data augmentation for a more robust dataset and employs MVT for classification. MVT processes input images by splitting them into patches and feeding them into a transformer in a sequence structure, similar to word embedding, followed by classification using a Multi-Layer Perceptron, this method aims to address challenges such as dataset imbalance and the need for comprehensive feature analysis in skin cancer classification (31).

Prior work has also examined fine-tuned transfer learning based on DenseNet121 for skin cancer classification, highlighting the strong baseline capability of DenseNet-based representations in dermoscopic image analysis (32–34). This supports the choice of DenseNet121 as the backbone in the present study, while our work extends this direction by integrating a diffusion-inspired Heat Flow Layer and transformer encoder components.

Our model, incorporating DenseNet121 with an Advanced Heat Flow Layer and transformer encoder layers, provides a unique approach in skin cancer classification, compared to other methods reviewed, which primarily focus on either CNNs or transformers, our model synergistically combines the strengths of both. This blend allows for nuanced feature extraction and spatial understanding, setting our approach apart in its ability to handle complex dermatoscopic image data, by integrating advanced image processing techniques and leveraging ensemble learning, our model aims to offer a more comprehensive and robust solution in skin lesion analysis.

Existing studies have explored CNNs, transformers, and hybrid CNN–Transformer models for skin lesion classification. In contrast, this work focuses on incorporating a diffusion-inspired intermediate processing layer within the classification pipeline and examining its empirical effect on feature quality and classification performance.

## 3 The proposed approach

Our skin cancer classification model is a sophisticated fusion of advanced image processing and machine learning techniques, central to this approach is the Advanced Heat Flow Layer, which implements an anisotropic diffusion process to enhance image features crucial for accurate classification. This layer, alongside the integration of a transformer encoder and DenseNet121 architecture, forms a robust and innovative model, the model aims to effectively capture both local and global features in dermatoscopic images, leveraging the strengths of convolutional and transformer-based neural networks for superior classification performance.

### 3.1 Advanced Heat Flow Layer

#### 3.1.1 Heat flow in image processing

In our machine learning model for skin cancer classification, we have incorporated a unique Heat Flow Layer that utilizes the concept of heat flow in image processing, drawing inspiration from the principles of thermodynamics, this layer simulates the physical phenomenon of heat diffusion through the image, guided by the heat equation (35), as shown in Equation 1:


$$\frac{\partial u}{\partial t}=\alpha \nabla^{2}u$$ (1)

Here, *u(x, t)* denotes the image intensity at a spatial coordinate *x* and time *t*, α represents the diffusion coefficient, and $\nabla^{2}u$ is the Laplacian, computing the second spatial derivative of image intensity, this foundation allows our Heat Flow Layer to conduct anisotropic diffusion, a process aiming to refine the image by smoothing out intensity variations, diminishing noise, and emphasizing critical features like edges, the rate and extent of diffusion are governed by α .

For practical computation in digital images, the continuous heat equation is discretized, we approximate the Laplacian $\nabla^{2}u$ through finite difference methods, in a two-dimensional image grid, this approximation for a pixel *(i, j)* is shown in Equation 2:


$$\nabla^{2}u\approx u(i-1,j)+u(i+1,j)+u(i,j-1)+u(i,j+1)-4u(i,j)$$ (2)

This discrete representation enables iterative pixel intensity updates, mirroring heat diffusion across the image and enhancing features vital for skin cancer classification, our computational methodology ensures efficient image preprocessing, a crucial step in our comprehensive skin cancer detection framework. The Advanced Heat Flow Layer in our skin cancer classification model is predicated on the principles of anisotropic diffusion, a method that judiciously smooths images while conserving the integrity of their edges, this layer’s functionality can be broken down into several key mathematical components, each contributing to its ability to enhance image features crucial for accurate diagnosis.

#### 3.1.2 Gradient calculation with Sobel operators

The layer begins by computing the image gradients, *dx* and *dy*, using Sobel operators, these operators are designed to approximate the gradient of the image intensity function. For an image *I*, the gradient at each pixel location *(i, j)* is determined by the difference in intensity values in its immediate neighborhood, as shown in Equations 3, 4:


$$\begin{array}{l} \mathrm{dx}(i,j)=(I(i+1,j-1)+2I(i+1,j)+I(i+1,j+1))-\\ \left(I(i-1,j-1)+2I(i-1,j)+I(i-1,j+1)\right) \end{array}$$ (3)

$$\begin{array}{l} \mathrm{dy}(i,j)=(I(i-1,j+1)+2I(i,j+1)+I(i+1,j+1))-\\ \left(I(i-1,j-1)+2I(i,j-1)+I(i+1,j-1)\right) \end{array}$$ (4)

These calculations are pivotal as they reveal the direction and intensity of the most significant changes in the image, forming the foundation for the subsequent diffusion process.

#### 3.1.3 Edge-stopping function for preserving image features

Following the gradient computation, an edge-stopping function *c* is derived, this function is essential for maintaining the distinct edges within the image during the smoothing process, it is defined using the magnitude of the gradient vector at each pixel, as shown in Equation 5:


$$c(i,j)=\exp(-\alpha \sqrt{\mathrm{dx}{\left(i,j\right)}^{2}+\mathrm{dy}{\left(i,j\right)}^{2}}$$ (5)

Here, *α* is a parameter fine-tuning the sensitivity of the function to the gradients, ensuring that the edges are preserved while the less significant variations in the image are smoothed out.

#### 3.1.4 Anisotropic diffusion

The core operation of the layer is the anisotropic diffusion process, which updates the image *x* iteratively, each update is governed by the calculated gradients and the edge-stopping function (16), the image at each iteration is updated, as shown in Equation 6:


$$x_{\mathrm{new}}(i,j)=x_{\mathrm{old}}(i,j)+\lambda \times \mathrm{div}(c.\mathrm{dx},c.\mathrm{dy})$$ (6)

Here, *λ* represents the diffusion rate, the divergence operator *div* in its discrete form is approximated as shown in Equation 7:


div(dx,dy)(i,j)=dx(i+1,j)−dx(i,j)+dy(i,j+1)−dy(i,j) (7)

This formula essentially calculates the net flow at each point, allowing for a controlled smoothing of the image that respects its inherent structural features.

#### 3.1.5 Efficient TensorFlow implementation

This entire process is implemented within the TensorFlow framework, leveraging its efficient computation capabilities, the Sobel filter constants and the divergence computations are optimized for parallel processing, ensuring that the layer operates seamlessly within the larger neural network architecture. In essence, the Advanced Heat Flow Layer employs a sophisticated blend of mathematical techniques to enhance the quality of images, this enhancement is crucial in the context of skin cancer detection, where the clarity and distinction of image features can significantly influence the accuracy of diagnosis.

#### 3.1.6 Discussion of stability and differentiability

The proposed Heat Flow Layer is implemented as a finite sequence of tensor operations, including Sobel filtering, pointwise arithmetic, exponential weighting, and iterative updates. These operations are differentiable within TensorFlow’s automatic differentiation framework, allowing the layer to be trained end-to-end through backpropagation.

In this work, the layer is used as a practical feature refinement module rather than as a strict numerical solver for a continuous PDE. Therefore, we do not claim a formal proof of convergence. Instead, numerical stability is maintained in practice by using a limited number of diffusion iterations and controlled diffusion parameters, which produced stable training behavior in our experiments.

In the current implementation, the diffusion coefficient *α* and the diffusion rate *λ* are treated as fixed hyperparameters selected empirically prior to training and are not updated during backpropagation.

### 3.2 Transformer Encoder Layer

The Transformer Encoder Layer in our neural network architecture is adeptly designed to capture and process complex data dependencies, this layer intertwines layer normalization, attention mechanisms, and feed-forward networks, each contributing uniquely to the overall functionality (36–38). Here, we unravel the mathematical intricacies embedded within this layer.

#### 3.2.1 Layer normalization

Initially, the layer undertakes the process of Layer Normalization, this step normalizes the input features across each feature dimension. For any given input feature *x_i_*, the normalized output y_i_ is calculated by subtracting the mean μ of the features and dividing by the square root of the variance $\sigma^{2}$ plus a small constant ϵ for numerical stability, the formula is expressed as shown in Equation 8:


$$y_{i}=\frac{x_{i}-\mu }{\sqrt{\sigma^{2}+\in }}$$ (8)

#### 3.2.2 Dimension expansion

Subsequent to normalization, the layer expands the dimensions of the data, this expansion adds a sequence length dimension, aligning the data structure to meet the requirements of the Multi Head Attention layer that follows.

#### 3.2.3 Multi-Head Attention mechanism

At the heart of the layer lies the Multi Head Attention mechanism, this mechanism enables the model to concurrently focus on different segments of the input sequence. It works on the principle of attention, where the output is a weighted sum of values, the weights assigned to each value are determined by a compatibility function of the query with the corresponding key, in mathematical terms, it is articulated as shown in Equation 9:


$$\text{Attention}\;(Q,,K,,V)=\text{softmax}(\frac{QK^{T}}{\sqrt{d_{k}}})\;V$$ (9)

The operation is replicated across multiple *heads* to diversify the model’s attentive capacity across various representation subspaces.

#### 3.2.4 Residual connection and dropout

Following the attention mechanism, the architecture integrates a residual connection and a dropout layer. The residual connection, which adds the input to the output of the attention layer, plays a vital role in alleviating the problem of vanishing gradients in deep networks. The dropout layer, on the other hand, acts as a regularizing element, randomly nullifying a portion of the output units to avert overfitting.

#### 3.2.5 Feed-forward network

Concluding the layer is a feed-forward network, consisting of two fully connected layers, between these layers, a *ReLU* activation function is employed. The first dense layer expands the dimensionality to *ff_dim*, and the second layer projects it back to the dimensionality of the original input, the feed-forward operation can be mathematically represented as shown in Equation 10:


$$\mathrm{FFN}(F)=\max(0,F.W_{1}).W_{2}$$ (10)

Where *F* is the output from the dropout layer, and *W_1_, W_2_* are the weights of the respective dense layers. In essence, the Transformer Encoder Layer is an amalgamation of carefully structured mathematical processes, each meticulously orchestrated to enhance the model’s capability to discern and interpret complex patterns in data, this capability is especially crucial in applications like skin cancer classification, where the precision and depth of data interpretation directly impact the accuracy of diagnosis.

### 3.3 CNN with attention and heat flow model

Our model for skin cancer classification intricately combines the principles of convolutional neural networks, advanced heat flow processing, and transformer-based attention mechanisms, this synergy of techniques is designed to offer a comprehensive analysis of dermoscopic images, crucial for accurate skin cancer detection.

The Advanced Heat Flow Layer starts by computing gradients *dx* and *dy* of the input image using Sobel filters, these filters, fundamental in edge detection, approximate the gradient in both the x and y directions, thus highlighting crucial features like edges and textures. The layer then engages in an anisotropic diffusion process, iteratively updating the image by applying diffusion influenced by the computed gradients and an edge-stopping function, this diffusion process is designed to enhance image features, particularly the edges, which are vital for skin lesion analysis. The model integrates DenseNet121, a dense convolutional network renowned for its efficient image feature extraction due to dense connections, the network processes the input image through its layers, each building upon the previous layers’ feature maps, an intermediate layer from DenseNet121 is selected for its rich representation of the input image, providing a comprehensive set of features for subsequent analysis.

Post processing through DenseNet121 and the Advanced Heat Flow layer, the resulting features are concatenated, this concatenation creates a fusion of refined heat flow features and the deep, hierarchical features extracted by DenseNet121, offering a robust representation of the image’s crucial characteristics, following the feature concatenation, the model employs additional convolutional layers, supplemented with Batch Normalization and ReLU activation, these layers further process the combined features, learning spatial hierarchies and enhancing feature representation, max Pooling layers are interspersed to reduce the spatial dimensions, which helps in achieving translational invariance and reducing the computational load.

Concatenation was selected as a simple and stable fusion strategy that preserves the full feature representations produced by both DenseNet121 and the Heat Flow Layer without introducing additional fusion parameters. This allows the subsequent convolutional layers to learn how to combine the complementary information from both branches. More sophisticated fusion strategies, such as residual fusion, attention-based fusion, or gating mechanisms, may further improve performance and can be investigated in future work.

**Figure 1:**
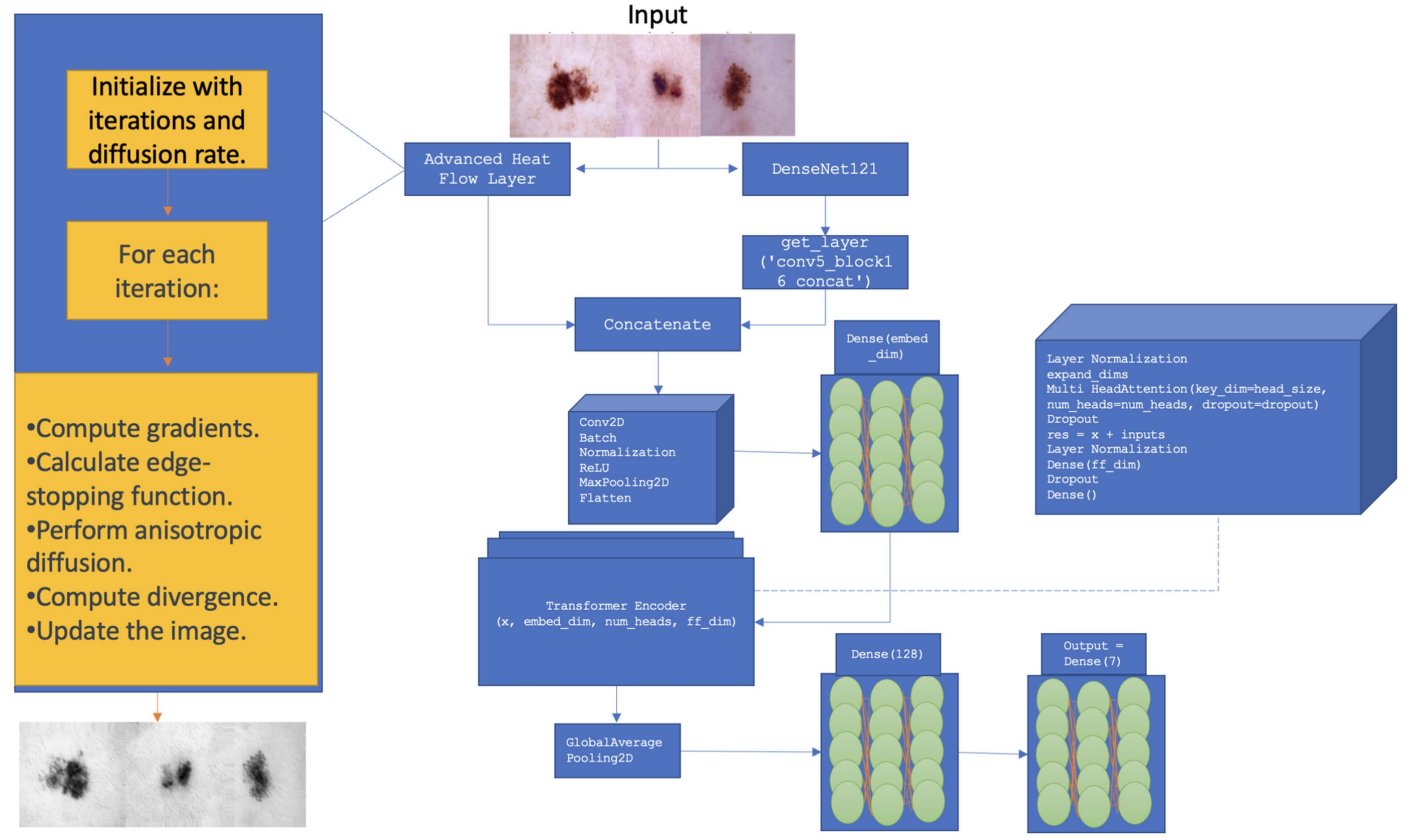
The proposed approach.

The model then transitions to incorporating Transformer encoder blocks, the flattened features from previous layers undergo Layer Normalization, stabilizing the training process. Multi-Head Attention within these blocks allows the model to attend to different parts of the input simultaneously, capturing intricate and complex dependencies, this is followed by a series of feed-forward networks comprising dense layers with ReLU activation, which process and refine the features further. In the final stage, Global Average Pooling is applied to condense the output of the Transformer blocks, focusing on the most significant features, the model concludes with a series of dense layers, culminating in a softmax layer that classifies the input into various categories indicative of different skin cancer types or benign conditions, the proposed approach is shown in Figure 1.

In essence, our model is a mathematical orchestration of various advanced techniques in image processing and machine learning, each step in the model, from initial feature enhancement using heat flow to the intricate pattern recognition capabilities of the Transformer blocks, is meticulously designed to extract, process, and interpret the complex nuances present in dermoscopic images, this comprehensive approach ensures that the model not only identifies a wide array of features within the images but also discerns the most critical elements for an effective and accurate diagnosis of skin cancer.

## 4 Experimental results

### 4.1 Experimental setup

We conducted comprehensive testing of our skin cancer classification model on the HAM10000 dataset, a well-known benchmark in dermatological image analysis, this dataset consists of 10,000 dermatoscopic images, providing a diverse and challenging test bed for model evaluation, our experiments were carried out on Google Colab Pro, utilizing a T4 GPU for efficient computation. To benchmark our model’s performance, we compared it against several state-of-the-art models: ViT-32, DarkNet, EfficientNet, InceptionV3, Xception, SqueezeNet, NASNetMobile, a CNN with attention mechanisms, Unet, ResNet, DenseNet121, and MobileNetV2, this comparison was designed to cover a broad spectrum of architectures, from traditional CNNs to more recent transformer-based models, ensuring a comprehensive evaluation of our model’s capabilities in the context of current advancements in the field. The HAM10000 dataset was divided into training and testing subsets using a ratio of [0.8, 0.2].

#### 4.1.1 ViT-32

The Vision Transformer model, specifically the ViT-32 variant, represents a significant shift in the approach to image classification tasks, unlike traditional convolutional neural networks, ViT-32 treats image classification as a sequence prediction problem, akin to models used in natural language processing. It processes images by dividing them into fixed-size patches, linearly embedding each of them, and then applying a series of Transformer layers, this approach allows ViT-32 to capture both local and global dependencies, leading to effective and efficient image classification performance (37).

#### 4.1.2 DarkNet

DarkNet, often associated with the YOLO (You Only Look Once) series of models, is a neural network framework known for its efficiency and speed, it is specifically designed for tasks requiring real-time processing, such as object detection and classification. DarkNet’s architecture is distinctively lightweight, enabling it to deliver high performance even on systems with limited computational resources, this makes it suitable for applications where rapid and efficient image processing is crucial, its ability to process images quickly, combined with reasonable accuracy, has made DarkNet a popular choice in various computer vision applications (39).

#### 4.1.3 EfficientNet

EfficientNet is a series of convolutional neural network models designed with efficiency in mind, both in terms of computational resources and model size, It is based on a scaled-up architecture that uniformly scales all dimensions of depth, width, and resolution, using a compound coefficient. This scaling method allows EfficientNet models to achieve higher accuracy and efficiency compared to other CNNs, the balance in scaling these dimensions is a key factor in its effectiveness, making EfficientNet particularly adept for tasks requiring high-performance image classification with constrained resource use (40).

#### 4.1.4 InceptionV3

InceptionV3 is a convolutional neural network model that is part of the Inception family, known for its architectural innovations in deep learning for image recognition tasks, this model introduces several upgrades from its predecessors, including the use of factorized convolutions and expanded the receptive field, which helps in efficient feature extraction at various scales. InceptionV3 is optimized for accuracy and computational efficiency, making it a popular choice in tasks requiring detailed image analysis. Its architecture allows for effective feature extraction with reduced computational costs, which contributes to its widespread use in various computer vision applications (41).

#### 4.1.5 Xception

Xception is a deep learning model renowned for its unique approach to convolutional neural networks, primarily through the implementation of depthwise separable convolutions, this architecture significantly improves upon the efficiency and performance of traditional CNNs. Xception stands out for its ability to process channels and spatial dimensions separately, leading to more effective feature extraction with reduced computational cost, this design not only enhances the model’s learning capability but also makes it more adaptable and efficient in handling complex image recognition tasks, particularly in the field of computer vision (42).

#### 4.1.6 SqueezeNet

SqueezeNet is a distinctive convolutional neural network architecture that demonstrates how smaller models can achieve accuracy comparable to larger networks, its design is characterized by the use of “squeeze” layers, which significantly reduce the number of parameters without compromising the performance. This makes SqueezeNet particularly efficient in terms of computational resources and storage, allowing it to be deployed in environments with limited capacity, Its small size, combined with its ability to maintain high accuracy, makes SqueezeNet a valuable model for real-time image processing applications and embedded systems (43).

#### 4.1.7 NASNetMobile

NASNetMobile is a convolutional neural network designed as part of the NASNet architecture, which was developed through neural architecture search (NAS), NASNetMobile, specifically, is optimized for mobile and embedded device applications, offering a balance between model size, computational efficiency, and accuracy. It is characterized by its scalability and flexibility, which allows it to adapt to different computational constraints while maintaining robust performance in image classification tasks, this makes NASNetMobile a suitable choice for applications where resources are limited but high accuracy is still required (44).

#### 4.1.8 CNN model with attention mechanisms

The CNN model with attention mechanisms we have developed integrates both squeeze-excite blocks and self-attention layers, the squeeze-excite blocks function by applying global average pooling, followed by a series of dense layers to create a channel-wise weighting. This process adaptively recalibrates channel feature responses, enhancing the representational capacity of the network. Meanwhile, the self-attention layers, implemented using MultiHeadAttention, facilitate the model’s ability to focus on relevant features within an image, improving its ability to discern complex patterns relevant to skin cancer classification, this combination of local feature recalibration and global contextual understanding positions our model for effective performance in skin lesion analysis.

#### 4.1.9 U-Net

U-Net is a convolutional neural network architecture that has gained prominence in the field of medical image analysis, particularly for tasks like image segmentation, its structure is characterized by a symmetric U-shaped design, facilitating precise localization combined with context information. U-Net’s unique feature is the use of skip connections, which connect layers in the contracting (downsampling) path to the expanding (upsampling) path, these connections help the network retain fine-grained details necessary for accurate segmentation, making U-Net highly effective in delineating intricate structures in medical images (45).

#### 4.1.10 ResNet

ResNet, short for Residual Network, is a groundbreaking convolutional neural network architecture that introduced the concept of residual learning to facilitate training of very deep networks, its distinctive feature is the use of “skip connections” or “shortcuts” to jump over some layers. These connections combat the vanishing gradient problem by allowing the flow of gradients directly through these shortcuts, With this architecture, ResNet can effectively learn from a significantly increased number of layers, enhancing its ability to capture complex features in images, which makes it highly effective for tasks like image classification (46).

#### 4.1.11 DenseNet121

DenseNet121, part of the Dense Convolutional Network (DenseNet) family, is a highly efficient convolutional neural network noted for its dense connectivity pattern, in DenseNet121, each layer obtains additional inputs from all preceding layers and passes on its own feature-maps to all subsequent layers. This architecture fosters feature reuse, reduces the number of parameters, and enhances feature propagation throughout the network, DenseNet121, with its 121 layers, is particularly effective in image classification tasks, providing a balance between depth and computational efficiency (14).

#### 4.1.12 MobileNetV2

MobileNetV2 is a significant advancement in the field of lightweight deep learning models for mobile and edge devices, it builds on the original MobileNet architecture, introducing inverted residual blocks with linear bottlenecks. These blocks enable the network to efficiently utilize the low-dimensional compressed representations, enhancing both accuracy and efficiency, MobileNetV2 is designed to be small and low-latency, making it ideal for applications with strict size and speed requirements, such as mobile phones and other embedded systems, without sacrificing performance on complex tasks like image classification (47).

### 4.2 Comparative analysis of model performance

Our developed model for skin cancer classification using the Ham10000 dataset has demonstrated good performance, surpassing other well-established models in various key metrics, as shown in Table 1. The incorporation of the Dense121 model as a backbone in our architecture is a strategic choice, leveraging its efficient feature extraction capability, however, the addition of the Advanced Heat Flow Layer is what sets our model apart, this layer applies anisotropic diffusion, enhancing the quality of the feature maps generated by Dense121. By doing so, it amplifies the model’s ability to distinguish between nuanced features of skin lesions, contributing to the increased precision and recall.

**Table 1:** The excremental results of our model compared to other models.

Models	Training loss	Training accuracy	Training precision	Training recall	Training F1 score	Training AUC	Testing loss	Testing accuracy	Testing precision	Testing recall	Testing F1 score	Testing AUC
ViT	0.915	0.672	0.78	0.573	0.66	0.928	0.86	0.684	0.814	0.578	0.676	0.937
DarkNet	0.028	0.99	0.99	0.989	0.99	0.99	1.679	0.729	0.74	0.727	0.733	0.9
efficientnet	1.15	0.66	0.66	0.66	0.66	0.87	1.13	0.67	0.67	0.67	0.67	0.88
InceptionV3	0.2	0.928	0.931	0.924	0.928	0.994	2.759	0.678	0.684	0.674	0.679	0.877
Xception	0.21	0.931	0.94	0.91	0.91	0.99	1.19	0.72	0.75	0.7	0.7	0.92
squeezenet	0.14	0.669	0.669	0.669	0.669	0.869	1.13	0.67	0.67	0.67	0.67	0.87
NASNetMobile	0.45	0.83	0.89	0.78	0.83	0.98	1.055	0.701	0.74	0.66	0.69	0.92
cnn_with_attention	0.835	0.692	0.845	0.593	0.697	0.94	0.815	0.694	0.8	0.637	0.713	0.944
Unet	0.073	0.977	0.978	0.975	0.977	0.998	1.696	0.752	0.759	0.749	0.754	0.92
Resnet	0.021	0.993	0.993	0.993	0.993	0.999	1.681	0.75	0.756	0.744	0.75	0.916
Dense121	0.056	0.982	0.984	0.978	0.981	0.999	2.744	0.751	0.755	0.748	0.751	0.9
MobileNetV2	0.222	0.918	0.927	0.907	0.917	0.99	1.44	0.731	0.744	0.72	0.735	0.921
Our Model	0.058	0.983	0.984	0.982	0.983	0.998	1.34	0.796	0.794	0.782	0.788	0.926

The effectiveness of the Heat Flow Layer is evident when comparing our model’s performance with the standalone Dense121 model, while Dense121 provides a robust foundation, our model refines and enhances its output through the Heat Flow Layer. This layer effectively smooths out irrelevant variations in the image while preserving critical structural details, leading to more accurate classification results, this precision is particularly crucial in medical imaging, where the distinction between benign and malignant lesions can be subtle. Besides the Heat Flow Layer, the integration of a transformer encoder plays a significant role in our model’s performance, the transformer encoder, with its layer normalization and multi-head attention mechanisms, allows the model to capture complex patterns in data, which is often missed by conventional CNNs, this ability to process long-range dependencies within the image further boosts the model’s classification capabilities, as evidenced by its superior metrics.

Our model’s superior performance can be attributed to the synergistic integration of the Dense121 backbone with the Advanced Heat Flow Layer and the transformer encoder, the Heat Flow Layer’s unique contribution in enhancing feature quality, combined with the transformer’s ability to capture complex patterns, results in a model that not only leverages the strengths of Dense121 but also significantly builds upon them, this innovative architecture and its empirical validation through enhanced performance metrics underscore our model’s effectiveness in skin cancer classification.

### 4.3 Metrics

Evaluating classification models involves key metrics:

1. Loss: It quantifies the discrepancy between predicted and actual values, commonly using mean squared error or cross-entropy loss.
2. Accuracy: Defined as the ratio of correct predictions to the total predictions made, it combines true positives and true negatives, as shown in Equation 11:

$$\text{Accuracy}=\frac{\mathrm{TP}+\mathrm{TN}}{\mathrm{TP}+\mathrm{TN}+\mathrm{FP}+\mathrm{FN}}$$ (11)

1. Precision: It gauges the correctness of positive predictions, indicating the fraction of true positives among all positive calls, as shown in Equation 12:

$$\text{Precision}=\frac{\mathrm{TP}}{\mathrm{TP}+\mathrm{FP}}$$ (12)

1. Recall: This measures the ability to identify true positives from all actual positives, as shown in Equation 13:

$$\text{Recall}=\frac{\mathrm{TP}}{\mathrm{TP}+\mathrm{FN}}$$ (13)

1. AUC: Reflecting overall model performance across thresholds, AUC compares true positive rates against false positive rates, with 1 indicating perfection and 0.5 showing no prediction skill.

The gap between training and testing performance indicates the presence of overfitting. For example, the proposed model achieves very high training accuracy while the testing accuracy is notably lower. This suggests that, although the model learns discriminative training features effectively, its generalization to unseen samples remains challenging. This limitation is common in medical imaging tasks with limited and imbalanced datasets and should be acknowledged when interpreting the reported results.

### 4.4 Heat Flow vs. Edge Detection Layers

To partially isolate the contribution of the proposed Heat Flow Layer, we compared it with a Sobel edge-based alternative across multiple DenseNet121 intermediate layers. Although this does not constitute a full component-wise ablation of every module in the architecture, it provides direct evidence regarding the effect of the custom image enhancement layer.

**Figure 2:**
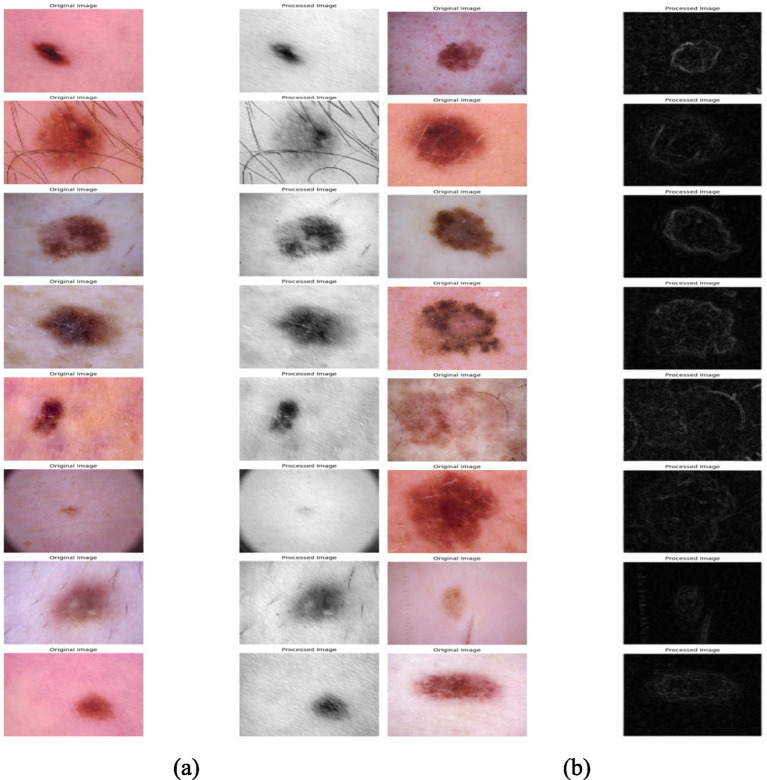
**(a)** Images after applying Heat Flow Layer, **(b)** images after applying Edge Detection Layer.

The Edge Detection Layer utilizes Sobel filters to detect edges in the images, it converts the input images to grayscale and then applies these filters to highlight the edges, this process emphasizes the contours and boundaries within the skin lesions, which are pivotal for accurate classification. Figure 2 shows images after applying the Heat Flow Layer and after applying the Edge Detection Layer.

We conducted experiments using different intermediate layers from Dense121: `conv3_block12_concat`, `conv4_block24_concat`, and `conv5_block16_concat`, for each, we compared the performance of the model using the Sobel Edges Layer against the model with the Heat Flow Layer. Table 2 shows the results.

**Table 2:** Comparing Sobel Edges Layer with Heat Flow Layer based on different intermediate layers.

Intermediate layer	Costume layer	Training loss	Training accuracy	Training precision	Training recall	Training F1 score	Training AUC	Training testing loss	Testing accuracy	Testing precision	Testing recall	Testing F1 score	Testing AUC
conv3_block 12_concat	Sobel Edges Layer	0.0462	0.986	0.987	0.986	0.986	0.99	1.86	0.747	0.753	0.744	0.748	0.906
	Heat Flow Layer	0.018	0.995	0.995	0.994	0.995	1	2.515	0.759	0.761	0.754	0.758	0.896
conv4_block 24_concat	Sobel Edges Layer	0.054	0.984	0.985	0.984	0.984	0.99	1.346	0.76	0.786	0.774	0.78	0.92
	Heat Flow Layer	0.03	0.992	0.993	0.992	0.992	0.99	2.351	0.776	0.793	0.782	0.788	0.917
conv5_block 16_concat	Sobel Edges Layer	0.057	0.984	0.985	0.983	0.98	0.99	1.34	0.779	0.795	0.784	0.79	0.925
	Heat Flow Layer	0.058	0.983	0.984	0.982	0.983	0.99	1.34	0.796	0.794	0.782	0.788	0.926

The comparative analysis reveals that while the Edge Detection Layer enhances the model’s ability to recognize lesion boundaries, it does not outperform the Heat Flow Layer in overall effectiveness, the Heat Flow Layer consistently demonstrates superior performance in terms of accuracy, precision, and generalization across different intermediate layers of Dense121. The effectiveness of the Heat Flow Layer, even over the Edge Detection Layer, can be attributed to its more comprehensive approach to image processing, while edge detection is valuable, the Heat Flow Layer’s anisotropic diffusion technique not only preserves edges but also enhances the overall quality of the feature maps. This results in better feature representation, which is crucial for the nuanced task of skin cancer classification, moreover, the results indicate that integrating these layers with different intermediate layers of Dense121 affects the performance. The `conv5_block16_concat` layer, in particular, seems to be the most effective point of integration, yielding the highest testing accuracy with both the Heat Flow Layer and the Edge Detection Layer, this suggests that later stages of Dense121, which capture more abstract representations, are more conducive to the additional processing offered by these layers. The experimental results underline the superiority of the Advanced Heat Flow Layer over the Edge Detection Layer in our CNN architecture, its ability to enhance feature representation while maintaining generalization is pivotal in achieving high accuracy and precision in skin cancer classification, additionally, the choice of the integration point within Dense121 significantly impacts the performance, with later layers like `conv5_block16_concat` proving to be more effective for layer integration.

While these comparisons provide useful partial ablation evidence for the proposed Heat Flow Layer, they do not fully isolate the individual contributions of all architectural components, such as DenseNet121, the transformer encoder, and their interaction. Therefore, the present analysis should be interpreted as targeted evidence for the effect of the custom enhancement layer rather than as a complete component-wise ablation of the full model.

### 4.5 Evaluation of augmented data on Advanced Heat Flow and Sobel Edge Detection Layers

To further validate the effectiveness of our model, we applied data augmentation techniques to the Ham10000 dataset, data augmentation is a crucial step in enhancing the robustness and generalizability of machine learning models, particularly in medical imaging, where the diversity of data is key to achieving high accuracy. This analysis serves as a partial ablation of the effect of training data variability on both layer choices. The data augmentation was implemented using TensorFlow’s `ImageDataGenerator`, the parameters for augmentation were carefully chosen to introduce variability without distorting the essential features of the skin lesions, the following augmentations were applied:

- Rotation range reduced to 10 degrees.
- Width and height shift ranges set to 0.1.
- Shear range adjusted to 0.1.
- Zoom range set to 0.1.
- Horizontal flipping enabled.
- Fill mode set to “nearest” for handling new pixel values.

This approach aimed to create a more diverse set of images that mimic real-world variations while maintaining the integrity of the diagnostic features in the skin lesions, the model was tested on the augmented dataset using the `conv5_block16_concat` intermediate layer from Dense121. Two configurations were evaluated: one with the Sobel Edges Layer and the other with the Advanced Heat Flow Layer. The results are shown in Table 3.

**Table 3:** Comparing Sobel Edges Layer with Heat Flow Layer after applying data augmentation.

Layer	Training loss	Training accuracy	Training precision	Training recall	Training F1 score	Training AUC	Testing loss	Testing accuracy	Testing precision	Testing recall	Testing F1 score	Testing AUC
Sobel Edges Layer	0.0242	0.912	0.924	0.903	0.913	0.994	0.553	0.824	0.84	0.814	0.826	0.97
Heat Flow Layer	0.1306	0.955	0.96	0.952	0.955	0.997	0.619	0.849	0.856	0.844	0.849	0.968

The results indicate that both layers improved their performance with augmented data, as seen in the increased testing metrics, however, the Heat Flow Layer outperformed the Sobel Edges Layer in every metric, confirming its superior capability in handling diverse and augmented datasets. The Heat Flow Layer’s advanced image processing techniques, which include anisotropic diffusion, allow it to adapt more effectively to variations introduced by augmentation, this results in higher accuracy, prec1ision, recall, and F1 scores, demonstrating its robustness in varied clinical scenarios. The Sobel Edges Layer, focusing primarily on edge detection, also showed improved performance, however, its relative simplicity compared to the Heat Flow Layer’s complex processing might limit its adaptability to the augmented variations. The experimentation with augmented data on the Ham10000 dataset reinforces the superiority of the Advanced Heat Flow Layer over the Sobel Edges Layer in skin cancer classification, the Heat Flow Layer’s ability to maintain high performance metrics, even with increased data variability, highlights its potential for clinical application in diverse and challenging diagnostic settings.

Similarly, although the augmented-data comparison helps clarify the robustness of the Heat Flow Layer relative to the Sobel-based alternative, it does not independently quantify the separate effects of the backbone network, transformer encoder, and augmentation strategy. A full ablation study examining each component individually would provide a more complete understanding of the performance gains and is left for future work.

### 4.6 Efficacy of the advanced Heat Flow Layer in skin cancer classification

The Advanced Heat Flow Layer represents a significant innovation in our skin cancer classification model, its effectiveness, particularly when compared to traditional smoothing operations in image processing, marks a breakthrough in medical image analysis. This subsection explains the reasons behind the superior performance of the Heat Flow Layer and its advantages over other smoothing techniques.

1. Anisotropic Diffusion: Unlike standard smoothing operations that uniformly blur the image, the Heat Flow Layer utilizes anisotropic diffusion, this process selectively smooths areas of an image while preserving crucial edge details. It achieves this by reducing noise and maintaining or enhancing the sharpness of important features like the boundaries of skin lesions.
2. Edge-Stopping Function: The layer employs a unique edge-stopping function, which is critical in preserving edge information, this function is based on the image gradients and ensures that the diffusion process does not blur the edges of lesions, a common drawback in standard smoothing techniques.
3. Adaptive Processing: The Heat Flow Layer adapts its processing based on the local structure of the image, it applies more diffusion in smoother regions while reducing diffusion near edges, this adaptability is particularly beneficial in medical imaging, where the distinction between normal and abnormal tissue can be subtle.
4. Gradient Computation with Sobel Filters: The use of Sobel filters for gradient computation further enhances the layer’s ability to detect and preserve edges, Sobel filters are effective in highlighting the most significant gradients, which correspond to edges and contours in medical images.

### 4.7 Comparison with traditional smoothing operations

Traditional smoothing operations, like Gaussian blurring, apply uniform smoothing across the image, while they are effective in reducing noise, they also tend to blur important features, such as the edges of lesions, This can lead to a loss of critical diagnostic information in medical images. In contrast, the Heat Flow Layer’s anisotropic diffusion preserves these vital features while still providing the noise reduction benefits of traditional smoothing, the results from our model testing clearly demonstrate the Heat Flow Layer’s superiority. When compared to other models employing different smoothing operations or layers like the Sobel Edges Layer, our model consistently showed higher accuracy, precision, and recall, the enhanced ability to preserve edge information while reducing noise significantly improves the model’s ability to accurately classify skin lesions, a key requirement in medical diagnostics. In the context of augmented data from the Ham10000 dataset, the Heat Flow Layer’s effectiveness was even more pronounced, it showed remarkable adaptability to the variations introduced by augmentation, maintaining high performance metrics, a testament to its robustness and reliability. The Advanced Heat Flow Layer, with its anisotropic diffusion mechanism and edge-preserving capabilities, clearly outperforms traditional smoothing operations in the context of skin cancer classification, its ability to adaptively smooth images while preserving crucial diagnostic features like lesion edges makes it a highly effective tool in medical image analysis.

## 5 Conclusion and future directions

Our study demonstrates a significant advancement in the field of dermatological diagnostics through the development of a deep learning model incorporating the Advanced Heat Flow Layer, this innovative approach has consistently outperformed benchmark models, including Vit-32, DarkNet, EfficientNet, InceptionV3, Xception, SqueezeNet, NASNetMobile, CNN with attention, Unet, Resnet, Dense121, and MobileNetV2, across various key performance metrics such as accuracy, precision, recall, F1 score, and AUC. Notably, our model achieved remarkable results, especially in terms of testing accuracy and precision, indicating its robustness and effectiveness in skin cancer classification tasks. The integration of the Advanced Heat Flow Layer, which employs anisotropic diffusion for enhancing image features while preserving essential details, has proven to be a crucial factor in the model’s success this layer effectively differentiates between benign and malignant lesions, a challenging aspect in medical imaging, by maintaining edge integrity and enhancing feature representation.

Looking ahead, several avenues for further research and development present themselves. First, expanding dataset diversity is essential to enhance the model’s generalizability. By incorporating images from a wider range of demographics and skin types, future work could address potential biases and improve the model’s applicability across diverse patient populations. Second, real-world clinical testing is crucial, as implementing and testing the model in clinical settings would provide valuable insights into its practical utility and areas for improvement, particularly regarding the user interface and integration with existing healthcare systems. Third, combining this model with other diagnostic modalities, such as dermoscopy and histopathological analysis, could create a more comprehensive diagnostic tool, potentially increasing the accuracy and reliability of skin cancer diagnoses. Fourth, enhancing the model’s interpretability is vital for clinical acceptance. Future efforts could focus on developing methods to visualize and explain the model’s decision-making process, thereby increasing trust and understanding among medical professionals. Finally, investigating the model’s adaptability through transfer learning to other forms of medical imaging and diseases could open up new applications in medical AI, leveraging the strengths of the Advanced Heat Flow Layer in various diagnostic challenges. Although the Heat Flow versus Sobel comparison and the augmented-data analysis provide partial ablation evidence, a full component-wise ablation separating the individual and joint effects of DenseNet121, the transformer encoder, the Heat Flow Layer, and data augmentation remains an important direction for future work.

In conclusion, our model represents a significant step forward in the application of deep learning to medical imaging, specifically in the challenging domain of skin cancer classification, the promising results pave the way for more advanced, accurate, and reliable diagnostic tools, potentially transforming the landscape of dermatological care and beyond.

## Data Availability

Publicly available datasets were analyzed in this study. This data can be found here: https://www.kaggle.com/datasets/kmader/skin-cancer-mnist-ham10000.
